# Mitral valve leaflet length as an important factor to differentiate hypertrophic cardiomyopathy from other causes of left ventricular hypertrophy

**DOI:** 10.1186/1532-429X-18-S1-P272

**Published:** 2016-01-27

**Authors:** Yong Luo, Dan Yang, Hong Liu, Ke Wan, Jiayu Sun, Tianjing Zhang, Yucheng Chen

**Affiliations:** 1 grid.412901.f0000000417701022Cardiology Department, West China Hospital, Chengdu, China; 2 grid.412901.f0000000417701022Radiology Department, Westchina Hospital, Chengdu, China; 3 Siemens MR Northeastern Collaboration, Beijing, China

## Background

Left ventricular hypertrophy (LVH) with Left ventricular maximal thickness (LVMT) ≥15 mm as a current criteria to differentiate hypertrophic cardiomyopathy (HCM) from other causes of LVH lacks specificity. The recent findings of intrinsic mitral valve leaflet elongation in HCM may potentially distinguish HCM from other causes of LVH. We aim to studying the performance of mitral valve leaflet length in differential diagnosis of HCM from other diseases with LVH.

## Methods

Contrast-enhanced cardiac magnetic resonance was performed following a standard protocol in 291 subjects (grouped by 112 HCM patients; 89 patients with LVH secondary to hypertension, cardiac amyloidosis or aortic stenosis; and 90 controls). Differences in anterior and posterior mitral leaflet length (AML and PML) (mm) among groups were analyzed, as were the diagnostic performance of AML or PML in distinguishing HCM from non HCM LVH.

## Results

AML and PML in HCM group were longest among the three groups (AML: HCM 26.49 ± 4.28 vs. non HCM LVH 21.98 ± 3.57 VS. normal 21.10 ± 3.12, p < 0.001; PML: HCM 13.57 ± 3.12 vs. non HCM LVH 11.47 ± 2.35 vs. normal 11.72 ± 2.89, p < 0.001). Furthermore, AML and PML were elongating only in HCM regardless of division by LVMT(<15 mm and ≥15 mm) or hypertrophic pattern (symmetric and asymmetric) (p < 0.05). Multiple logistic regression analysis showed AML, PML and LVMT were predictors to differentiate HCM from non HCM LVH with LVMT≥15 mm (p < 0.05). When performed ROC, LVMT>21 mm was proved an optimal cutoff value to differentiate HCM from non HCM LVH with LVMT≥15 mm (AUC 0.779 [95%CI 0.708-0.839]; sensitivity 61.6%, specificity 85.71%). In patients with LVMT between 15 mm and 21 mm, AML> 24 mm and PML>13 mm showed similarly favorable differential diagnosis value between HCM and non HCM LVH by ROC curves (AML: AUC 0.761 [95%CI 0.660-0.844], sensitivity 67.4%, specificity 81.2%; PML: AUC 0.737 [95%CI 0.634-0.823]; sensitivity 51.2%, specificity 89.6%).

## Conclusions

Elongation of mitral leaflet length in HCM is a specific and intrinsic characteristic and it has a favorable differential value to discriminate HCM from non HCM diseases.

